# Two machine learning-based nomogram to predict risk and prognostic factors for liver metastasis from pancreatic neuroendocrine tumors: a multicenter study

**DOI:** 10.1186/s12885-023-10893-4

**Published:** 2023-06-09

**Authors:** Jianbo Li, Long Huang, Chengyu Liao, Guozhong Liu, Yifeng Tian, Shi Chen

**Affiliations:** 1 Shengli Clinical Medical College of Fujian Medical University, Fujian Medical University, Fuzhou, 350001 China; 2 Department of Hepatobiliary Pancreatic Surgery, Fujian Provincial Hospital, Fuzhou, 350001 China; 3 Department of Hepatopancreatobiliary Surgery, First Affiliated Hospital of Fujian Medical University, Fujian, 350005 China

**Keywords:** Pancreatic neuroendocrine tumor, Liver metastasis, Machine learning, Nomogram, Surveillance Epidemiology and End Results (SEER) database

## Abstract

**Background:**

Pancreatic neuroendocrine tumors (PNETs) are one of the most common endocrine tumors, and liver metastasis (LMs) are the most common location of metastasis from PNETS; However, there is no valid nomogram to predict the diagnosis and prognosis of liver metastasis (LMs) from PNETs. Therefore, we aimed to develop a valid predictive model to aid physicians in making better clinical decisions.

**Methods:**

We screened patients in the Surveillance, Epidemiology, and End Results (SEER) database from 2010–2016. Feature selection was performed by machine learning algorithms and then models were constructed. Two nomograms were constructed based on the feature selection algorithm to predict the prognosis and risk of LMs from PNETs. We then used the area under the curve (AUC), receiver operating characteristic (ROC) curve, calibration plot and consistency index (C-index) to evaluate the discrimination and accuracy of the nomograms. Kaplan-Meier (K-M) survival curves and decision curve analysis (DCA) were also used further to validate the clinical efficacy of the nomograms. In the external validation set, the same validation is performed.

**Results:**

Of the 1998 patients screened from the SEER database with a pathological diagnosis of PNET, 343 (17.2%) had LMs at the time of diagnosis. The independent risk factors for the occurrence of LMs in PNET patients included histological grade, N stage, surgery, chemotherapy, tumor size and bone metastasis. According to Cox regression analysis, we found that histological subtype, histological grade, surgery, age, and brain metastasis were independent prognostic factors for PNET patients with LMs. Based on these factors, the two nomograms demonstrated good performance in model evaluation.

**Conclusion:**

We developed two clinically significant predictive models to aid physicians in personalized clinical decision-makings.

**Supplementary Information:**

The online version contains supplementary material available at 10.1186/s12885-023-10893-4

## Introduction

Pancreatic neuroendocrine tumors (PNETs) are rare and clinically heterogeneous tumors that originate from neuroendocrine cells of the digestive and respiratory systems [1]. The pancreas is one of the most common sites for endocrine tumors [2]. In recent years, the incidence of pancreatic neuroendocrine tumor (PNET) in the population has been on the rise with the development of diagnostic techniques and the increasing awareness of endocrine tumors. Approximately 28%-77% of PNET patients develop liver metastasis (LMs) [3]. In the past, PNETs were considered inert, but recent studies have found that some PNETs are highly aggressive [4, 5]. LMs are a common site of distant metastasis from PNETs [6] and have a devastating impact on the health of patients with PNETs. By analyzing the risk factors, we can provide an early diagnosis of LMs in patients with PNETs. The ability to accurately predict the survival of patients with hepatic metastasis from PNETs will enable doctors to better conduct clinical management. The prognostic factors for overall survival (OS) in PNET patients with hepatic metastasis are controversial [7, 8]. Our aim is to investigate the prognostic factors for OS in PNET patients with hepatic metastasis and the risk factors for hepatic metastasis from PNETs and to construct a clinically important nomogram.

Nomograms are widely used for tumor prognosis and recurrence widely, mainly because they allow visualization of statistical prediction models that provide intuitive numerical evaluation of the possibility of incidents (e.g. death or recurrence) [9, 10]. For PNET, TNM stage, a classic indicator, still has advantages for pathological staging [11]. For many cancers, the nomogram grading approach is superior to conventional staging systems and has become a new standard [12, 13].

To our knowledge, no models have been developed to predict the occurrence of LMs from PNETs and OS in patients with LMs from PNETs Therefore, we aimed to construct and validate a nomogram using clinical data from a large multicenter sample and use it to predict whether PNETs develop LMs as well as the 1-year, 2-year and 3-year OS of patients with LMs from PNETs.

## Methods

### Patient selection

Data on patients diagnosed with PNETs from 2010–2016 were screened from the Surveillance, Epidemiology, and End Results (SEER) database, which contains the demographic, clinicopathological and follow-up information of the populations from 18 medical centers in the United States. Because the SEER database, which collects the tumor-related information of approximately 30% of the entire United States population, has incomparable data from individual centers, the results can be better extrapolated to the general population than studies conducted at a single center. We collected data for patients who met the inclusion criterion of PNET as the primary cancer. The exclusion criteria were as follows: (1) incomplete follow-up data; (2) unclear cause of death; and (3) unknown features. A total of 1998 patients diagnosed with PNETs were included in study, including 343 patients with LMs. In addition, retrospective study was conducted at the First Hospital of Fujian Medical University and Fujian Provincial Hospital, and data on patients with PNETs were collected from 2012–2021 for external validation. The inclusion and exclusion criteria for the external validation cohort were consistent with those for the internal cohort. The study was approved by the Ethics Committee of Fujian Provincial Hospital and the First Hospital of Fujian Medical University.

### Data elements

We used SEER*Stat software version 8.4.0 to retrieve data from the SEER database (SEER Study Data, 18 registry, November 2019 Sub - 2000–2017) for our study. The original location of pancreatic tumor was listed as C25.0 ~ C25.9 according to site and morphology. The following histological/behavioural codes according to the International Classification of Diseases of Oncology, Third Revision (ICD-O-3) were uesd: 8150/3: pancreatic endocrine tumor, malignant; 8151/3: insulinoma,malignant;8152/3: glucagonoma,malignant;8153/3:gastrinoma,malignant;8155/3:vipoma,malignant;8156/3:somatostatinoma, malignant;8240/3: carcinoid tumor, NOS;8242/3: enterochromaffin-like cell tumor, malignant; 8243/3:goblet cell carcinoid;8246/3:neuroendocrine carcinoma, NOS; and 8249/3:atypical carcinoid tumor. Patient statistics included age at diagnosis, race, gender, marital status, histologic subtype, grading, tumor size, primary site, TNM stage, surgery at primary site (from RX Summ-Surg Prim site (1998 +)), lymph nodes removed in surgery, radiotherapy, chemotherapy, bone metastasis, brain metastasis and lung metastasis. In our survival analysis, the outcome variable was OS, regarded as the time from diagnosis to death or the last follow-up. Using an electronic medical record system, we collected clinical medical information from patients with PNETs for the external validation cohort.

### Statistical analysis

For all statistical analyses, SPSS (version 25.0) and R software version 4.10 were used. For statistical methods, continuous variables are represented as the interquartile ranges (IQR) or median (extreme deviation), and nonnormally distributed continuous variables were compared using the Mann-Whitney U test. Frequency data are presented as numbers, and comparisons between frequency data were assessed using the chi-square test or Fisher’s exact test. Significant features were identified from the available features by using machine learning algorithms, such as least absolute shrinkage and selection operator (Lasso) regression, which deals with multicollinearity in the available features, and random forest, which screens variables for selection based on the effect of the variable on the prediction of the outcome. Factors predicting LMs from PNETs were then identified by univariate and multivariate logistic regression analyses. The strength of the association between risk factors and LMs in patients with PNETs was assessed by calculating the odds ratios (ORs) and 95% confidence intervals (CIs). Factors significantly associated with PNETs were identified in feature selection and machine learning algorithms were incorporated into the multivariate analysis. The SPSS Optimal Binning method was used to select optimal cut-off points for continuous variables. The composition of the bins is optimized according to the classification wizard variables of the “supervised” binning process, and some discrete variables could be considered as categorical variables in the process of differentiating them.

A nomogram based on the results of machine learning algorithms and univariable logistic analysis was constructed to predict the probability of LMs in patients with PNETs. We then analysed the survival of 343 PNET patients with LMs to determine their prognostic factors. There were 245 patients in the training group and 98 patients in the internal validation group according to the a 7:3 ratio. We performed univariate Cox regression analysis on all variables, and recursive feature elimination for feature selection. With reference to recursive feature elimination and multiple Cox regression, information loss was minimized by the Akaike information criterion (AIC). Therefore, we identified independent prognostic factors for LMs from PNETs. Overall, two risk prediction models based on risk factors and independent prognostic factors were developed to predict the risk and OS of PNET patients. The consistency index (C-index) and receiver operating characteristic (ROC) methods were used to evaluate the accuracy of nomogram and calibration curve were used to validate its discrimination. Decision curve analysis (DCA) is a method to assess the clinical utility of different prediction models [14]. By quantifying the net benefit at different threshold probabilities, nomogram models can be compared with other models. As DCA can display false positive and true positive scores as a function of the risk threshold, it compensates for the shortcomings of the ROC curve [15]. In our research, *P* < 0.05 (two-sided) was considered statistically significant.

## Results

### Patient characteristics

In our study, 1998 patients with PNETs were contained based on our inclusion and exclusion criteria. Of these, 343 patients had LMs from PNETs, with 233 in the training cohort and 110 in the internal validation cohort. Table 1 shows the baseline clinical characteristics and treatment options for patients. For statistical purposes, continuous variables were categorized according to the best classification method, with statistically significant differences in marriage (*P* < 0.01), and pathological subtypes (*P* < 0.01) between those without LMs and those with LMs. The most common histological subtype was carcinoid tumor (41.69%).

**Table 1:** Baseline clinical features and treatment regimen of pancreatic neuroendocrine tumor patients

	**Without Liver metastasis number (*n* = 1655)**	**With Liver metastasis number (*n* = 343)**	***P*-value**
Age			0.875*
Median	55.87	55.65	
Range	10–85	15–85	
Marital status			0.009
No married	1090	214	
Married	547	147	
Sex			
Female	759	159	0.448
Male	878	202	
Histology			< 0.001
Pancreatic endocrine tumor	85	15	
Insulinoma	20	0	
Glucagonoma	3	1	
Gastrinoma	4	2	
Vipoma	1	0	
Somatostatinoma	2	0	
Carcinoid tumor	754	79	
Goblet cell carcinoid	1	0	
Neuroendocrine carcinoma	712	240	
Atypical carcinoid tumor	75	24	
Grade			< 0.001
Well differentiated; Grade I	1252	164	
Moderately differentiated; Grade II	301	96	
Poorly differentiated; Grade III	61	71	
Undifferentiated; anaplastic; Grade IV	23	30	
Primary Site			< 0.001
Head of pancreas	486	120	
Body of pancreas	273	27	
Tail of pancreas	599	140	
Others	279	74	
AJCC T stage			< 0.001
T0	1	3	
T1	627	16	
T2	556	127	
T3	403	160	
T4	50	55	
AJCC N stage			< 0.001
N0	1273	179	
N1	364	182	
Surgery			
No	203	208	< 0.001
Yes	1434	153	
Surgery Lymph Node			
No	461	218	< 0.001
Yes	1176	143	
Radiotherapy			
No	1599	327	< 0.001
Yes	48	34	
Chemotherapy			
No	1528	179	< 0.001
Yes	109	182	
Tumor size			
0–26 mm	869	42	< 0.001
26–36 mm	122	147	
> 36 mm	646	940	
Brain metastasis			
No	1636	358	0.200
Yes	1	3	
Lung metastasis			
No	1627	338	< 0.001
Yes	10	23	
Bone metastasis			< 0.001
No	1631	338	
Yes	6	23	

^*^Mann-Whitney U test

Among the primary sites, PNETs occurred most frequently in the tail of the pancreas (36.99%). T2 (34.18%) and N0 (72.67%) were most common T and N stages, and PNETs of size 36–180 mm (79.38%) were most common. For treatment, 1587 patients (79.68%) underwent surgery, 82 patients (4.10%) were treated with radiotherapy and 291 patients (14.56%) were treated with chemotherapy. There were 4 patients (0.33%) of brain metastasis, 33 patients (1.65%) of lung metastasis and 29 patients (1.45%) of distant metastasis from PNETs.

### Diagnostic nomogram model establishment and validation

**Fig. 1:**
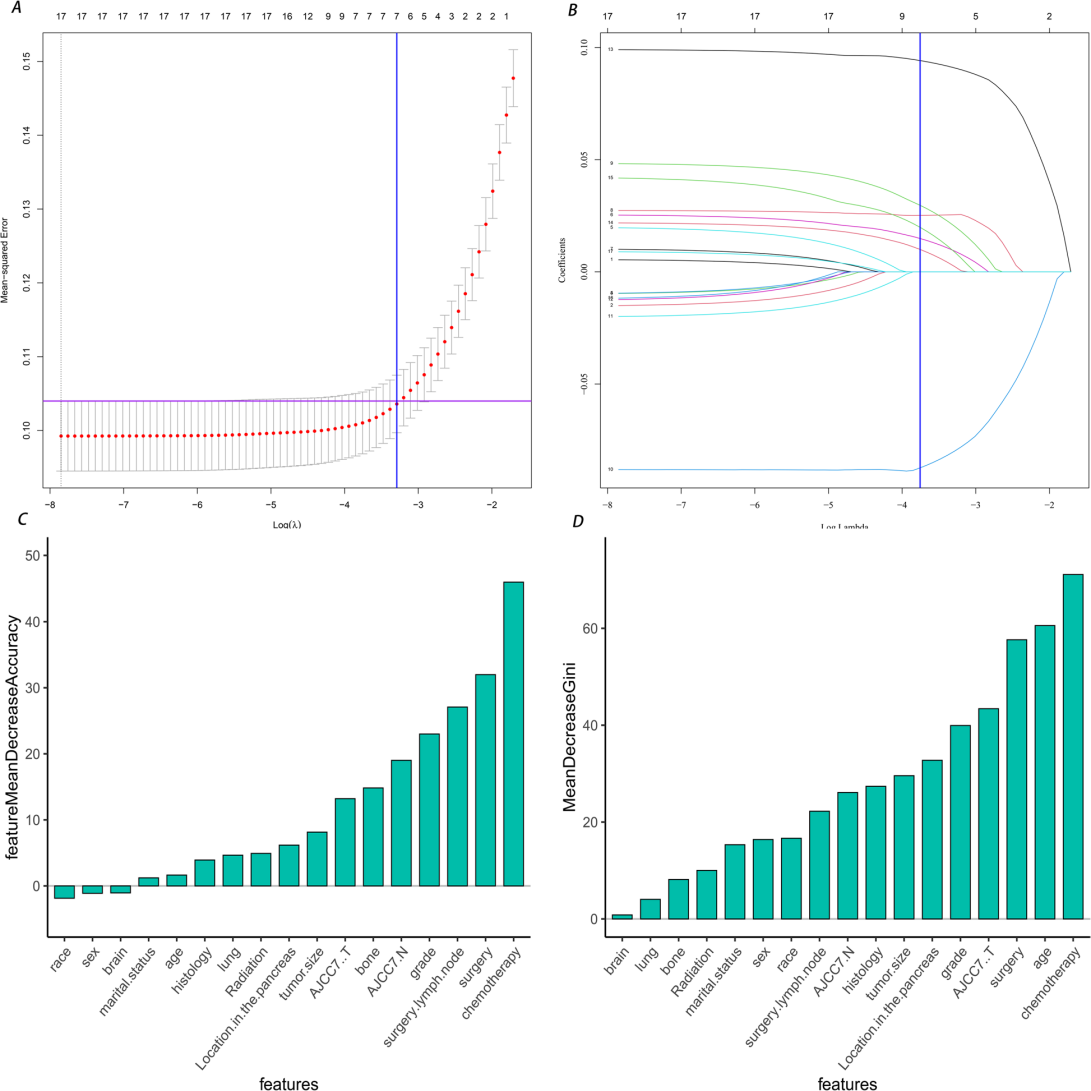
Predictor selection using the least absolute shrinkage and selection operator (Lasso) logistic regression model and Ranking of input variables in the random forest model to predict pancreatic neuroendocrine tumors. **A** Identification of the optimal penalization coefficient lambda (λ) in the Lasso model used tenfold cross-validation and the minimum criterion. **B** Lasso coefficient profiles of the 17 clinical features. The dotted vertical line was plotted at the value selected using tenfold cross-validation in (**A**), for which the optimal λ resulted in 7 non-zero coefficients. **C** Mean decrease in accuracy. **D** Mean decrease in Gini. Variables are listed from most important to least important based on the mean decrease in accuracy and mean decrease in the Gini coefficient

First, we used the Lasso algorithm to select the feature variables and obtained eight feature variables (Fig. 1A-B). Second, seven characteristic variables were identified to predict LMs from PNETs by the random forest method (Fig. 1C-D), which were all included in the variables screened by the Lasso algorithm. Finally, six variables (tumor grade, chemotherapy, bone metastasis, tumor size, AJCC N stage, and surgery) were screened by combining univariate and multivariate analyses (Table 2).

**Table 2:** Univariable and multivariable logistic regression of risk factor of liver metastasis in pancreatic neuroendocrine tumor patients

**Characteristics**	**Univariate analysis**		**Multivariate analysis**	
	**OR (95%CI)**	***P*-value**	**OR (95%CI)**	***P*-value**
Age	0.999 (0..991–1.007)	0.780		
Marital status				
No married	Reference		Reference	
Married	0.731 (0.578–0.923)	0.008	0.813 (0.609–1.091)	0.167
Sex				
Female	Reference			
Male	0.911 (0.724–1.145)	0.423		
Histology				
Pancreatic endocrine tumor	Reference			
Insulinoma	0.721 (0.349–1.490)	0.377		
Glucagonoma	0.000 (0.000–0.000)	0.998		
Gastrinoma	1.042 (0.103_10.488)	0.972		
Vipoma	1.563 (0.269–9.068)	0.619		
Somatostatinoma	0.000 (0.000–0.000)	1.000		
Carcinoid tumor	0.000 (0.000–0.000)	0.999		
Goblet cell carcinoid	0.327 (0.196–0.548)	0.000		
Neuroendocrine carcinoma	0.000 (0.000–0.000)	1.000		
Atypical carcinoid tumor	1.053 (0.650–1.706)	0.833		
Grade				
Well differentiated; Grade I	Reference		Reference	
Moderately differentiated; Grade II	2.435 (1.837–3.227)	< 0.001	1.171 (0.695–1.975)	0.553
Poorly differentiated; Grade III	8.888 (6.083–12.979)	< 0.001	1.631 (1.015–2.620)	0.043
Undifferentiated; anaplastic; Grade IV	9.958 (5.648–17.556)	< 0.001	0.813 (0.565–1.170)	0.266
Primary Site				
Head of pancreas	Reference		Reference	
Body of pancreas	0.423 (0.274–0.657)	< 0.001	0.760 (0.439–1.314)	0.760
Tail of pancreas	1.056 (0.805–1.386)	0.692	2.076 (1.446–2.981)	< 0.001
Others	1.135 (0.828–1.558)	0.432	1.330 (0.865–2.040)	0.191
AJCC T stage				
T0	Reference		Reference	
T1	3.541 (1.918–7.349)	< 0.001	22.566 (1.711–297.667)	0.180
T2	4.351 (2.354–8.763)	< 0.001	0.451 (0.198–1.076)	0.073
T3	1.435 (0.783–2.948)	0.074	1.008 (0.585–1.737)	0.978
T4	2.727 (0.275–27.076)	0.240	1.525 (0.879–2.846)	0.133
AJCC N stage				
N0	Reference		Reference	
N1	1.2686 (1.032–1.506)	< 0.001	2.348 (1.724–3.197)	< 0.001
Surgery				
No	Reference		Reference	
Yes	0.1041 (0.081–0.134)	< 0.001	0.156 (0.113–0.214)	< 0.001
Surgery Lymph Node				
No	Reference		Reference	
Yes	3.889 (3.069–4.928)	< 0.001	0.546 (0.329–0.906)	0.019
Radiotherapy				
No	Reference	< 0.001	Reference	
Yes	2.036 (1.136–3.648)		1.591 (0.877–2.888)	0.144
Chemotherapy				
No	Reference		Reference	
Yes	0.530 (0.359–0.782)	0.001	0.272 (0.191–0.385)	0.609
Tumor size				
0-26 mm	Reference		Reference	
26-36 mm	1.009 (1.006–1.013)	< 0.001	2.201 (1.183–4.098)	0.013
> 36 mm	2.224 (1.629–3.049)	< 0.001	3.859 (2.628–5.668)	< 0.001
Brain metastasis				
No	Reference		Reference	
Yes	13.7090 (1.422–132.180)	0.023	4.968 (0.334–73.900)	< 0.001
Lung metastasis				
No	Reference		Reference	
Yes	11.071 (5.221–23.475)	< 0.001	1.153 (0.435–3.058)	0.775
Bone metastasis				
No	Reference		Reference	
Yes	18.498 (7.475–45.774)	< 0.001	9.048 (2.785–29.400)	< 0.001

**Fig. 2:**
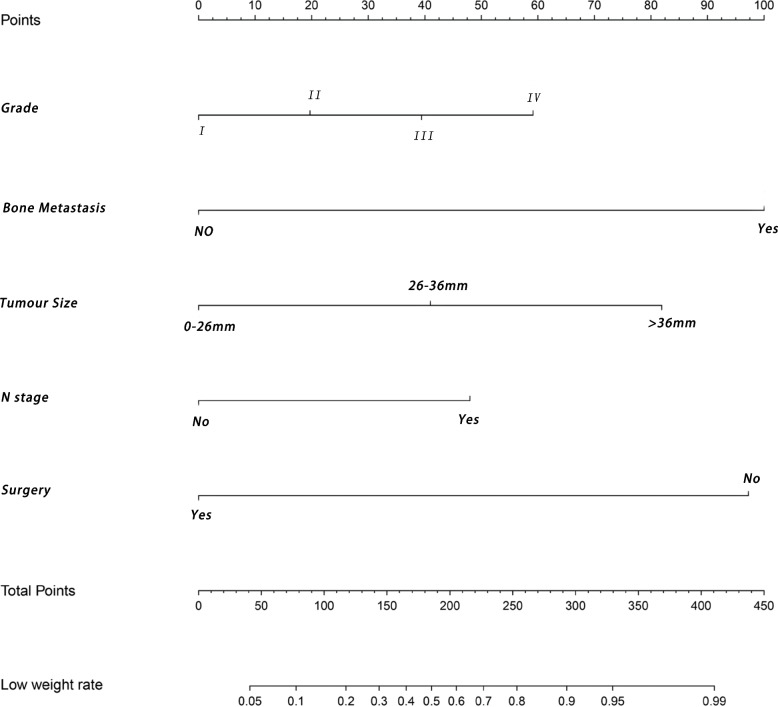
Nomogram to estimate the risk of liver metastasis in patients with pancreatic neuroendocrine tumors

**Fig. 3:**
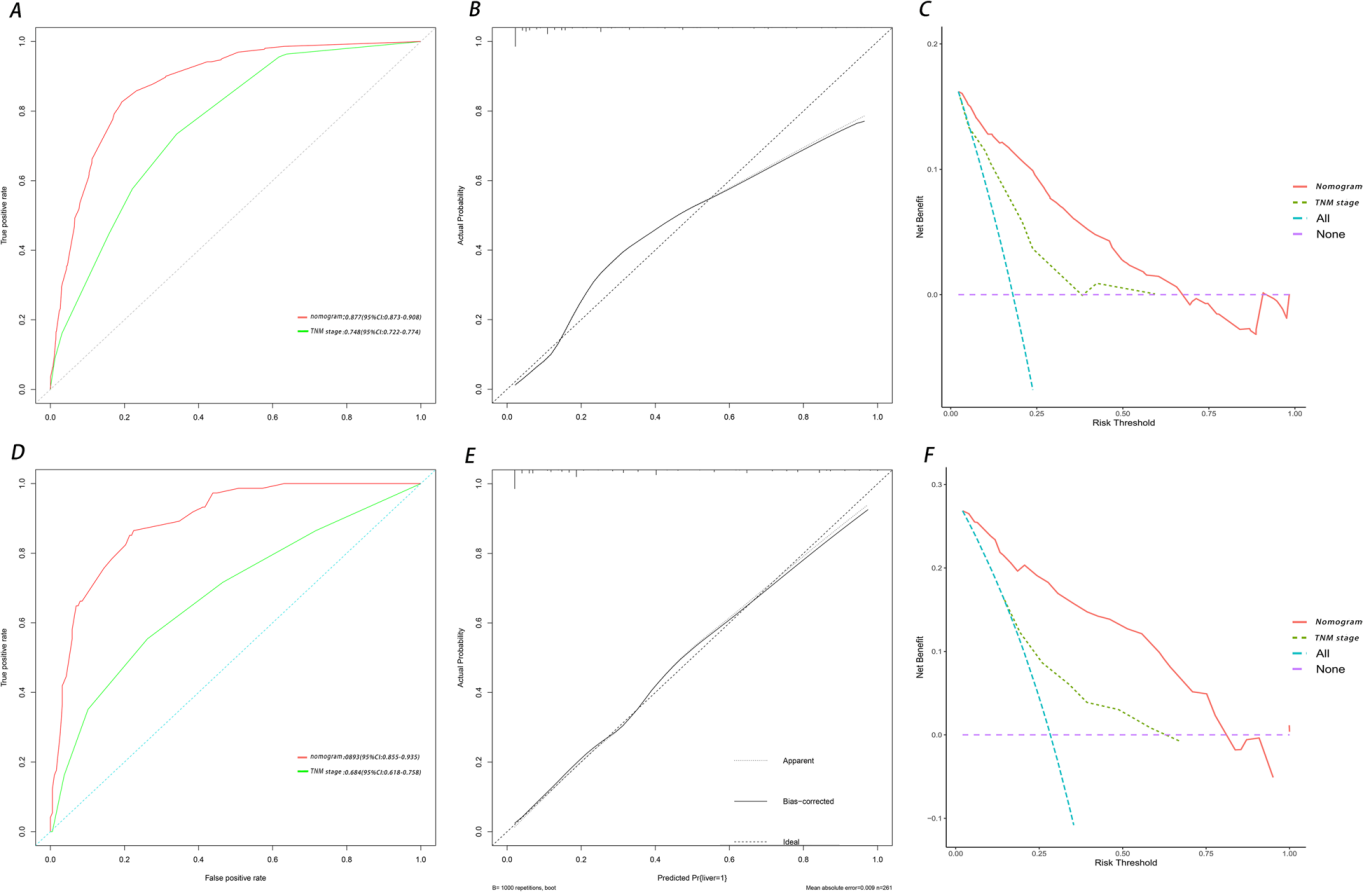
Comparison of the area under the receiver operating characteristic curves, calibration plots and DCA curves between the nomogram and TNM stage in the training cohort (**A**-**C**) and external validation cohort (**D**-**F**)

The independent predictors obtained by feature selection was used to construct a nomogram model for predicting the risk of LMs from PNETs (Fig. 2). ROC analysis showed that the nomogram had an area under the curve (AUC) value of 0.877, signifying that the model has excellent accuracy (Fig. 3A). The calibration curves indicated that nomogram performed well (Fig. 3B). Furthermore, the DCA indicated that the nomogram model was valid for clinical application (Fig. 3C). To further validate the model in the Chinese population, an external validation cohort was created and the corresponding evaluation curve was plotted. ROC analysis demonstrated an AUC value of 0.893 for the nomogram in the external validation cohort, indicating that the model also has good discriminatory power in the Chinese population (Fig. 3D). The calibration curve and DCA (Fig. 3E-F) showed that the nomogram model performed better in clinical practice. We also plotted the ROC and DCA curves for TNM staging, which showing that the new nomogram outperformed TNM staging in terms of discrimination.

### Prognostic nomogram model establishment and validation

**Fig. 4:**
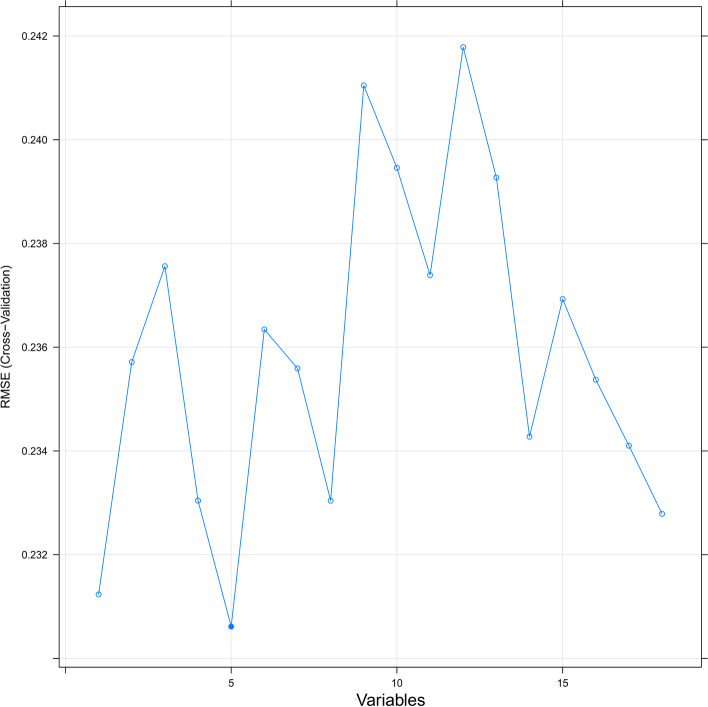
The random forest model was constructed iteratively, the best evaluated feature was selected, and the root mean square error (RMSE) was minimized when the random forest included 5 variables

**Fig. 5:**
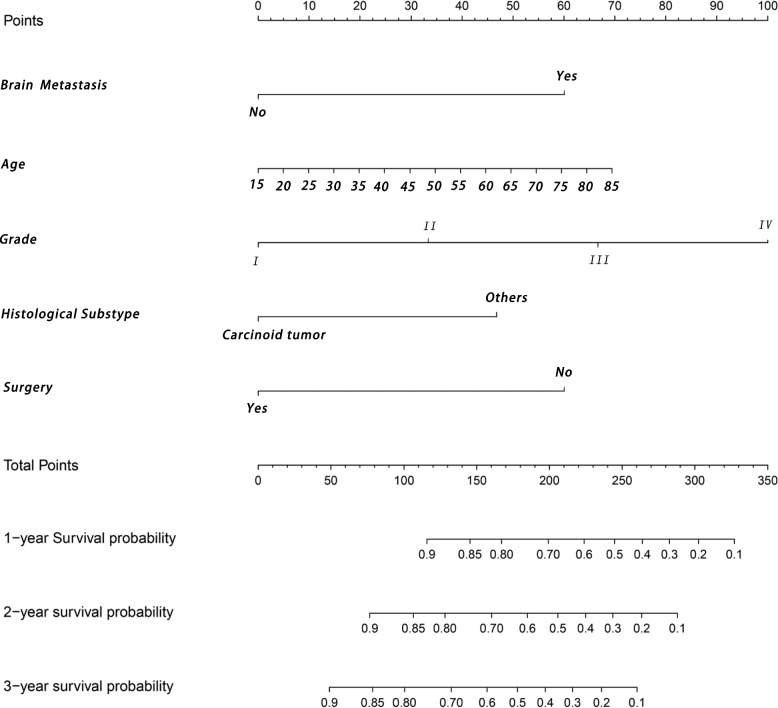
Nomogram for predicting the overall survival of patients with pancreatic neuroendocrine tumors presenting with liver metastases. To use this nomogram, the specific point for each variable of the patient lies on each variable axis. Draw a vertical line upwards to determine the points of which each variable; the sum of these points is located on the total points axis, and draw a vertical line down to the survival axis to determine the probabilities of 1-, 2- and 3- year overall survival

The clinical characteristics and treatment options of patients with PNETs and LMs are shown in Table 3. The chi-square test, the Mann-Whitney U test and Fisher’s exact test indicated that all variables were not significantly different between the training and validation cohorts. In the training cohort, the variables were screened univariate Cox regression, and recursive feature elimination with the AIC, those screened variables were included in the multivariate Cox regression, which showed that histological grade, N stage, surgery, chemotherapy, tumor size, and bone metastasis were factors affecting prognosis (*P* < 0.05) (Table 4). Variables with *P* < 0.05 were included in multivariate Cox regression analysis, combined with a random forest-based feature recursive elimination method (Fig. 4) to screen the final variables for inclusion in the model. Based on the screened variable, we constructed a prognostic nomogram model for PNETs (Fig. 5).

**Table 3:** Demographic and clinicopathological characteristics in pancreatic neuroendocrine tumor patients with liver metastasis

**Characteristics**	**Training cohort**		**Validation cohort**		**χ**^**2**^	***P***
	**n**	**%**	**n**	**%**		
Age						0.939*
Median	55.65		55			
Age	20–85		15–85			
Marital status					1.570	0.210
No married	101	42.4%	37	35.2%		
Married	137	57.6%	68	64.8%		
Sex					0.116	0.733
Female	109	45.8%	46	43.8%		
Male	59	54.2%	59	56.2%		
Histology					1.498	0.221
^a^Carcinoid tumor:	66	27.7%	36	34.3%		
^b^Others	172	72.3%	69	65.7%		
Gade					4.512	0.211
Well differentiated; Grade I	105	44.1%	57	54.3%		
Moderately differentiated; Grade II	69	29.0%	25	23.8%		
Poorly differentiated; Grade III	44	18.5%	19	18.1%		
Undifferentiated; anaplastic; Grade IV	20	8.4%	4	3.8%		
Primary Site					2.428	0.489
Head of pancreas	81	34.0%	9	8.6%		
Body of pancreas	15	6.3%	31	29.5%		
Tail of pancreas	91	38.2%	18	17.1%		
Others	51	21.4%	47	44.8%		
AJCC T stage					3.419	0.490
T0	1	0.4%	2	1.9%		
T1	9	3.8%	7	6.7%		
T2	84	35.3%	36	34.3%		
T3	109	45.8%	44	41.9%		
T4	35	14.7%	16	15.2%		
AJCC N stage					0.861	0.354
N0	114	47.9%	55	53.3%		
N1	124	52.1%	49	46.7%		
Surgery					0.012	0.912
No	132	55.9%	58	55.2%		
Yes	105	44.1%	47	44.8%		
Surgery Lymph Node					0.133	0.716
No	141	59.2%	60	57.1%		
Yes	91	40.8%	45	42.9%		
Radiotherapy					0.103	0.749
No	215	90.3%	96	91.4		
Yes	23	9.7%	9	8.6%		
Chemotherapy					0.030	0.863
No	118	48.6%	51	48.6%		
Yes	120	50.4%	54	51.45		
Tumor size					2.430	0.297
0–26 mm	14	5.9%	11	10.5%		
26–36 mm	41	17.2%	19	18.1%		
> 36 mm	183	76.9%	75	71.4%		
Brain metastasis					0.060	0.807
No	218	91.6%	97	92.4%		
Yes	20	8.4%	8	7.6%		
Lung metastasis					3.046	0.081
No	227	95.4%	95	90.5%		
Yes	11	4.6%	10	9.5%		
Bone metastasis					0.175	0.676
No	224	94.1%	100	95.2%		
Yes	14	5.9%	5	4.8%		

^*^T test

^a^Carcinoid tumor: Atypical carcinoid tumor, Carcinoid tumor

^b^Others: Pancreatic endocrine tumor, Insulinoma, Glucagonoma, Gastrinoma, Vipoma, Somatostatinoma, Goblet cell carcinoid, Neuroendocrine carcinoma

**Table 4:** Univariate and multivariate Cox proportional hazards regression analysis in pancreatic carcinoma patients with bone metastasis

**Characteristics**	**Univariate analysis**		**Multivariate analysis**	
	**OR (95%CI)**	***P***	**OR (95%CI)**	***P***
Age	0.982 (0.966–0.999)	0.041	1.301 (0.958–1.765)	0.092
Marital status				
No married	Reference			
Married	1.355 (0.842–2.180)	0.211		
Sex				
Female	Reference			
Male	0.923 (0.581–1.485)	0.733		
Histology				
^a^Carcinoid tumor:	Reference		Reference	
^b^Others	2.274 (1.366–3.784)	0.002	1.979 (1.179–3.322)	0.010
Gade				
Well differentiated; Grade I	Reference		Reference	
Moderately differentiated; Grade II	1.498 (0.856–2.622)	0.157	1.512 (0.915–2.497)	0.106
Poorly differentiated; Grade III	1.257 (0.671–2.354)	0.475	2.343 (1.340–4.146)	0.003
Undifferentiated; anaplastic; Grade IV	0.517 (0.297–0.899)	0.019	4.596 (2.343–9.015)	< 0.001
Primary Site				
Head of pancreas	Reference		Reference	
Body of pancreas	0.755 (0.327–1.742)	0.096	0.902 (0.363–2.245)	0.825
Tail of pancreas	1.447 (0.827–2.529)	0.510	1.416 (0.808–2.248)	0.247
Others	1.038 (0.597–1.804)	0.412	1.475 (0.754–2.847)	0.352
AJCC T stage				
T0	Reference			
T1	2.023 (0.493–8.298)	0.244		
T2	0.740 (0.265–2.062)	0.497		
T3	1.238 (0.802–1.911)	0.328		
T4	1.489 (0.866–2.559)	0.679		
AJCC N stage				
N0	Reference			
N1	1.103 (0.754–1.613)	0.615		
Surgery				
No	Reference		Reference	
Yes	0.391 (0.261–0.587)	< 0.001	0.412 (0.263–0.646)	< 0.001
Surgery Lymph Node				
No	Reference		Reference	
Yes	0.493 (0.331–0.734)	< 0.001	2.166 (0.737–6.368)	0.160
Radiotherapy				
No	Reference		Reference	
Yes	2.036 (1.136–3.648)	0.017	1.613 (0.881–2.954)	0.121
Chemotherapy				
No	Reference		Reference	
Yes	0.530 (0.359–0.782)	0.001	0.906 (0.582–1.411)	0.662
Tumor size				
0–26 mm	Reference			
26–36 mm	1.955 (0.648–5.895)	0.234		
> 36 mm	0.543 (0.356–1.163)	0.144		
Brain metastasis				
No	Reference		Reference	
Yes	1.654 (0.906–3.017)	0.099	2.205 (1.111–4.375)	< 0.001
Lung metastasis				
No			Reference	
Yes	2.971 (1.626–5.432)	< 0.001	1.241 (0.587–2.619)	0.572
Bone metastasis				
No	Reference			
Yes	1.540 (0.588–4.037)	0.380		

^a^Carcinoid tumor: Atypical carcinoid tumor, Carcinoid tumor

^b^Others: Pancreatic endocrine tumor, Insulinoma, Glucagonoma, Gastrinoma, Vipoma, Somatostatinoma, Goblet cell carcinoid, Neuroendocrine carcinoma

**Fig. 6:**
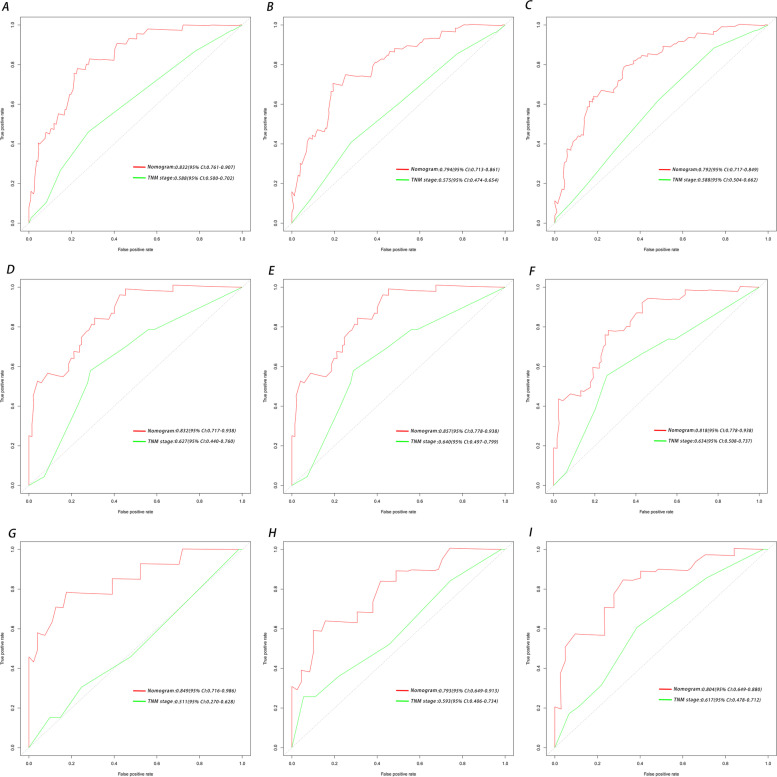
ROC curves of the ability of the nomogram and TNM stage to predict 1-, 2- and 3-year overall survival in the **A**–**C** training cohort, **D**–**F** internal validation cohort, and **G**–**I** external validation cohort

The C-index values were 0.752 (95% CI: 0.694–0.795) in the training set, 0.740 (95% CI: 0.657–0.823) in the internal validation cohort, and 0.760 (95% CI: 0.657–0.823) in the external validation cohort. The nomogram performed relatively well in terms of its discrimination ability through the area AUC and DCA. The AUCs of the Cox regression model for predicting 1-year OS, 2-year OS, and 3-year OS in the training, internal validation, and external validation sets were 0.832 (95% CI: 0.761–0.907), 0.794 (95% CI: 0.713–0.861) and 0.792 (95% CI: 0.717–0.949), 0.832 (95% CI: 0.717–0.939), 0.857 (95% CI: 0.778–0.938) and 0.818 (95% CI: 0.716–0.980), 0.849 (95% CI: 0.716–0.980), and 0.793 (95% CI: 0.649–0.913), and 0.804 (95% CI: 0.706–0.880) respectively (Fig. 6).

**Fig. 7:**
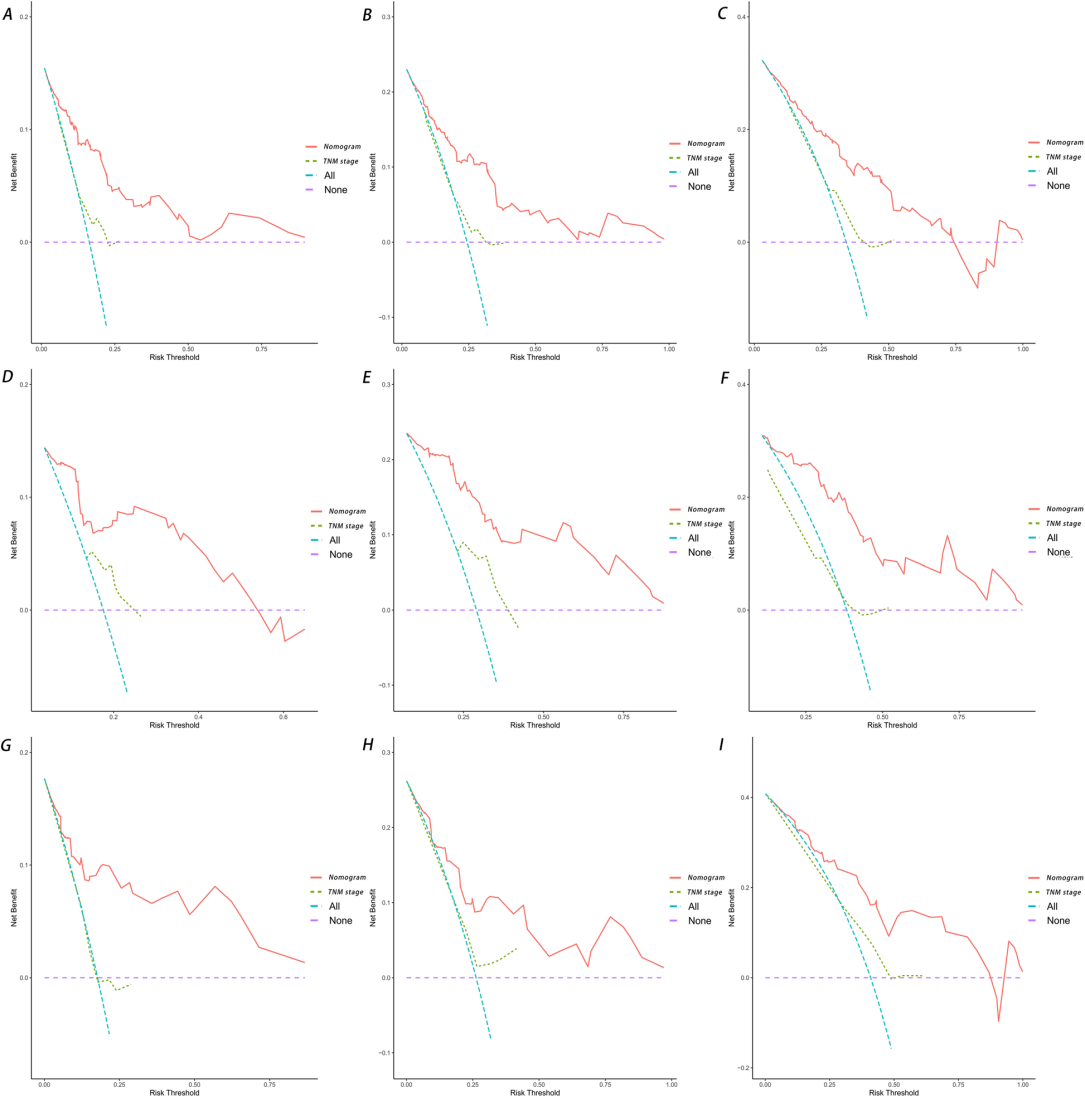
Decision curve analysis of the nomogram and TNM stage for the survival prediction of patients with pancreatic neuroendocrine tumors presenting with liver metastases. **A** 1-, 2- and 3-year survival benefits in the training cohort. **B** 1-,2- and 3-year survival benefits in the internal validation cohort. **C** 1-, 2- and 3-year survival benefits in the external validation cohort

**Fig. 8:**
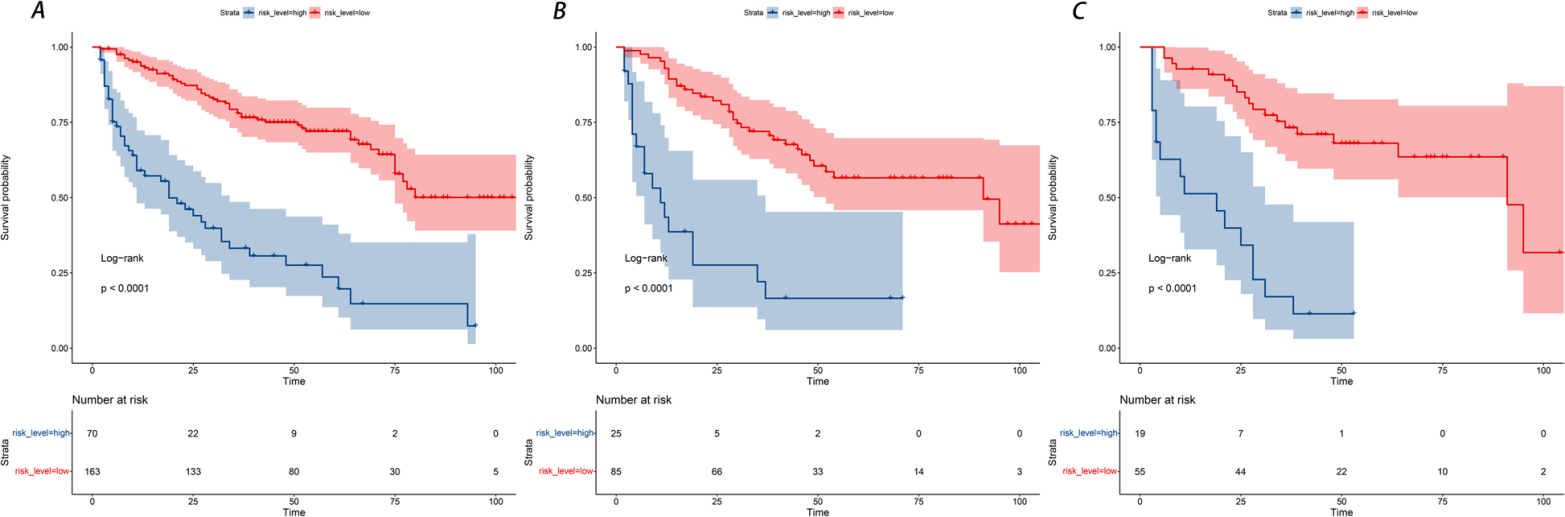
Kaplan-Meier curves of OS for patients in the low-risk and high-risk groups. **A** The training cohort; **B** the internal validation cohort; **C** the external validation cohort

In addition, DCA is widely used in the assessment of the clinical value of nomograms. As shown in Fig. 7, the net benefit of the nomogram mortality risk was significantly positive and superior to that of the conventional TNM staging system, indicating that the nomogram has significant clinical utility in predicting the OS of patients with LMs from PNETs. The training, internal validation, and external validation cohorts showed significant differences in survival rates according to Kaplan-Meier analysis (Fig. 8).

## Discussion

As the most common distant metastasis from PNETs, LMs accounts for the majority of patients with distant metastasis from PNETs and have an important effect on prognosis in PNETs; therefore, the identification of risk factors and prognostic factors for LMs from PNETs is important. In recent years, studies on LMs from PNETs have focused on treatment, mainly surgical modalities [7, 8, 16]. The most research to date involves case reports and single-cohort studies [17–19]. There are no studies on predicting the occurrence of LMs from PNETs and the prognosis after LMs; therefore, we performed a large sample size study retrospective study on risk factors and prognostic factors for LMs in patients in patients with PNETs and created a predictive nomogram.

We used machine learning algorithms and logistic regression analysis to explore the factors associated with PNETs at diagnosis. In addition, we used machine learning algorithms and Cox risk regression analysis to obtain survival estimates. Logistic analysis revealed that histological grade, N stage, surgery, chemotherapy, tumor size, and bone metastasis were independent factors in the diagnosis of LMs from PNETs. Using Cox risk regression analysis, we found that age, histological subtype, histological grade, surgery, and brain metastasis were independent prognostic factors for LMs in patients with PNETs. We found that age at diagnosis had no effect on the development of LMs in patients with PNETs. This finding is not consistent with studies of distant metastasis from other gastrointestinal tumors, such as pancreatic cancer, which have shown that age is an independent risk factor for distant metastasis [20–22]. In LMs from PNETs, age acts as a prognostic factor. As age increases, senescent cells accumulate in the tumor microenvironment, and cytokines secreted by senescent cells stimulate tumor proliferation and tumor trophoblast angiogenesis [23, 24]. We suspect that it is this effect that accelerates progression in older patients with hepatic metastasis from PNETs. There are also some studies in other diseases that suggest that senescence may affect the remodelling of the extracellular matrix of cells, leading to tumorigenesis and metastasis [25, 26], so the age as a factor in PNETs still needs further investigation. In our study, carcinoid tumors (30.0%) were the predominant subtype in patients with hepatic metastasis from PNETs. In addition, the carcinoid tumor subtype was a significant protective independent predictor of survival in patients with LMs from PNETs. This is reflected in other literature, where the protective effect of carcinoid tumors as inert tumors in PNETs may be due to the differential expression of the transcription factor PAX8.27 [27]. In PNETs, bone metastasis are rarer, unlike LMs and lung metastasis. Interestingly, although bone metastasis are a risk factor for LMs in patients with PNETs, they have no significant effect on the survival of patients with LMs from PNETs, and further research is needed to determine the exact mechanism. It is recommended that immediate and effective measures should be taken by oncologists to prevent PNET patients from developing distant metastasis, and they should also pay attention to whether bone metastasis occur in PNET patients after LMs.

As the database only contains the TNM stage of tumors and there is no WHO pathological classification, the TNM staging of PNETs was used in our study, which uses tumor size, proliferative features, lymph node metastasis and distant metastasis to assess disease status. The TNM stage is a classic, clinically proven and long-established clinical staging system [28, 29], whereas the WHO pathological classification is a histologic grade classification based on Ki-67 expression and mitotic counts, which describes the proliferative activity of the tumors and was adopted by the World Health Organization (WHO) [30]. In contrast to WHO pathological classification, which focuses on the proliferative potential of the tumor, TNM stage reflects the growth and metastasis of the tumor, can be observed preoperatively by non-invasive imaging, and is more readily available than the pathological stage of PNETs, which are advantages of using TNM stage as a clinical predictor. Somatostatin analogs (SSA) and targeted therapy are clinically effective for progressive PNENs. SSA acts by occupying the somatostatin receptor, is frequently expressed in both nonfunctioning and functioning tumors, and functions successfully mainly on symptomatology. In one study, tumor progression in mid- to late-stage, somatostatin inhibitor receptor-positive patients is effectively blocked by lanreotide, providing good symptom control [31] Molecular target therapy is based on gene mutations widely found in PNETs. Everolimus delays disease progression in chemotherapy-resistant patients with PNETs [32]. Both targeted therapy and SSA are effective treatments for intermediate to advanced PNENs, but we were unable to include these data in our analysis due to the limitations of the database available to us. In this investigation, Cox univariate regression showed that radiotherapy may lead to a poorer prognosis in patients with LMs from PNETs. In the guidelines, it was shown that there is a lack of evidence from large clinical trials on the efficacy of radiotherapy for PNETs, and we speculate that this may be due to a statistical bias in patients who are given radiotherapy because they have reached the end stage of the disease.

Compared to traditional methods, models constructed using machine learning algorithms for feature selection may achieve better predictive power across a variety of disease conditions [15]. More specifically, the use of machine learning methods significantly improved the accuracy of screening for risk factors in the prediction of the occurrence of LMs in patients with PNETs, outperforming traditional methods. Additionally, nomograms are widely used in modern medicine, particularly in the field of oncology [33, 34]. To validate the applicability of the models, internal and external validation was used for validation. To better ensure model generalization, it is not enough to evaluate the model only in an internal verification set. This problem is solved more effectively by the verification in an external verification set. We used an external validation set to test the generalization of the model. In addition, we took into account the differences between countries and regions and selected two large teaching hospitals for our external dataset to reduce possible selection bias. Surprisingly, the predictive performance of the model was not only good in the internal validation set, but also surprisingly good in the external validation set. Thus, the clinical features of our study could be of interest to oncologists in the context of PNET patients to better determine the progression of their disease and to guide clinical management.

Neverthless, Our study also has some limitations. First, the tumor grade in the SEER database differs from the current guidelines, which are based on mitotic counts and Ki-67 expression. Additionally, noninclusion of pathological staging classification for PNEN, which plays an important role in their initial management in the clinic, may have a large impact on the model. Second, this study was based on the SEER database, which contains limited information on the treatment and lacks detailed information about some key clinical biochemical indicators and treatment options, such as specific metastasis to lymph nodes, surgical procedures, blood insulin, SSA, target therapy and chemotherapy. Third, the SEER database has inaccurate information on histological subtypes; for example, the nomenclature of in PNETs has changed, and carcinoid tumors are well-differentiated and moderately differentiated PNETs, but the SEER database has not been updated. Finally, as a retrospective study, there may be some uncontrolled biases. In future research, we plan to include targeted therapy, SSA, molecular diagnostic parameters and tumor grading according to WHO and ENETS in our investigation of PNETs.

## Conclusion

To our knowledge, this is the first study to diagnose and predict the development of LMs in PNETs and the prognosis of patients with LMs with the largest sample size for now. The diagnosis nomogram for LMs prediction for patients with PNETs, and the corresponding survival prediction nomogram we proposed behave excellent predictive performance, which further aid clinicians in identifying high-risk patients and selecting appropriate treatment options.

## Supplementary Information

**Additional file 1: Supplementary Figure 1.** Flowchart of patients identified in this study.

**Additional file 2: Supplementary Figure 2.** Calibration curves of the nomograms. Calibration curves of 1-, 2- and 3-year overall survival for patients with pancreatic neuroendocrine tumors presenting with liver metastases in the (A–C) training cohort, (D–F) internal validation cohort, and (G–I) external validation cohort. The grey line represents the ideal reference line, where the predicted probability would match the observed survival rate. The blue dots are calculated by bootstrapping (resamples: 1000) and represent the nomogram performance. The closer the solid red line is to the dotted line, the more accurate the model is in predicting overall survival.

## Data Availability

The datasets generated and/or analysed during the current study are available in the SEER database (http://seer.cancer.gov) repository. All data generated or analysed during this study are included in this published article and its supplementary information files.
