# Supplementary figures and images for: Two machine learning-based nomogram to predict risk and prognostic factors for liver metastasis from pancreatic neuroendocrine tumors: a multicenter study
Source: BMC Cancer. 2023 Jun 9;23:529. doi: 10.1186/s12885-023-10893-4 (PMC10257274; doi:10.1186/s12885-023-10893-4)

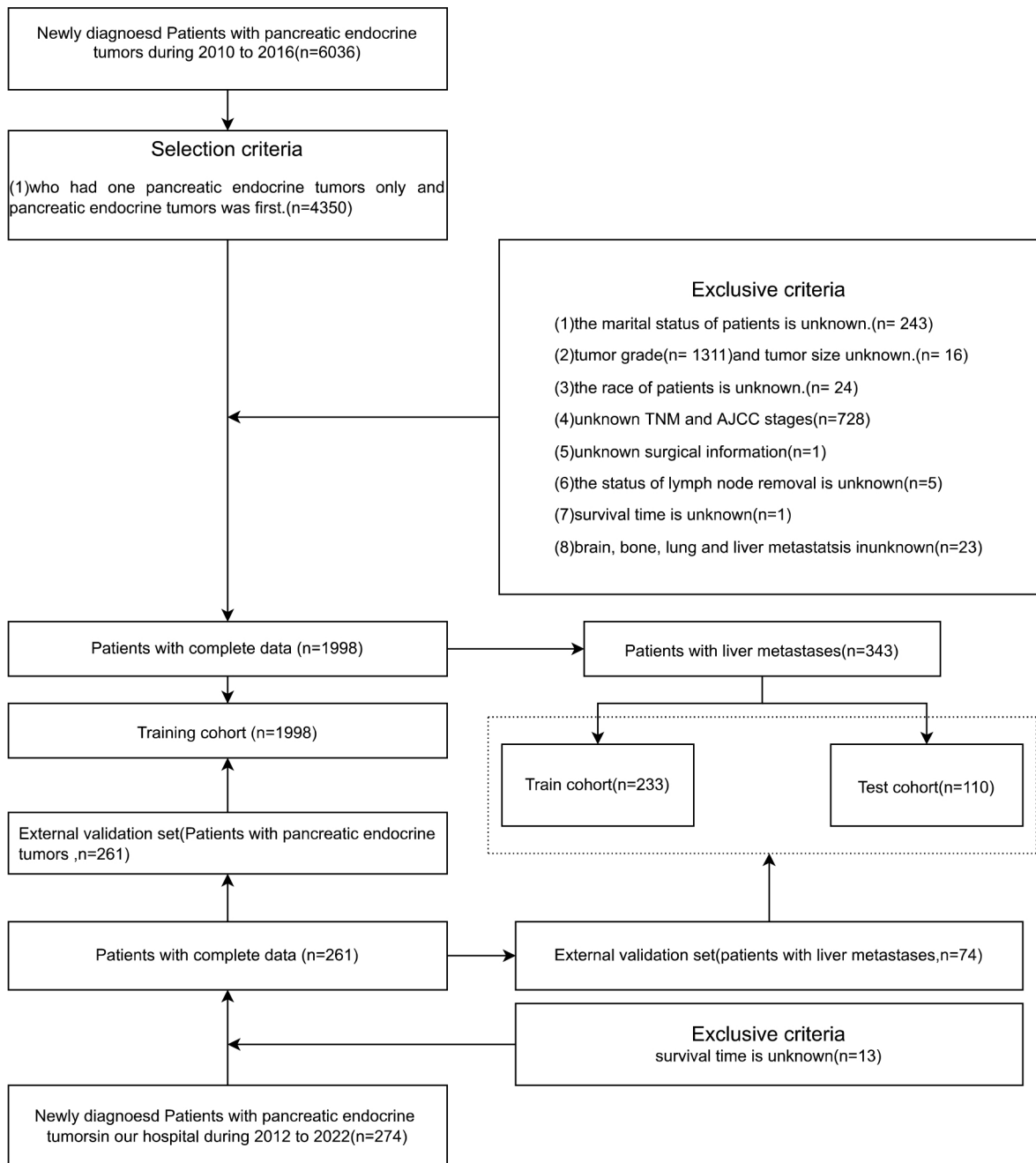

Supplement: Supplementary file 1 — Additional file 1: Supplementary Figure 1. Flowchart of patients identified in this study. [file 12885_2023_10893_MOESM1_ESM.pdf]

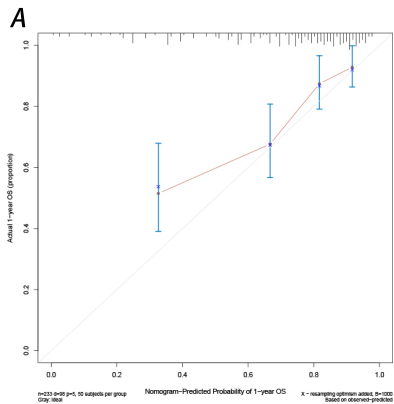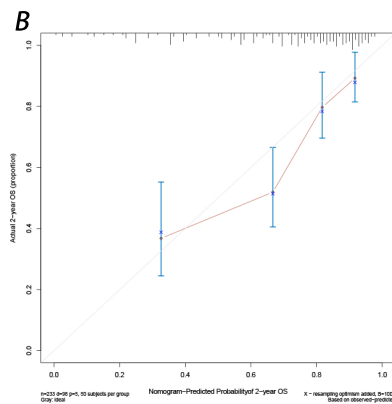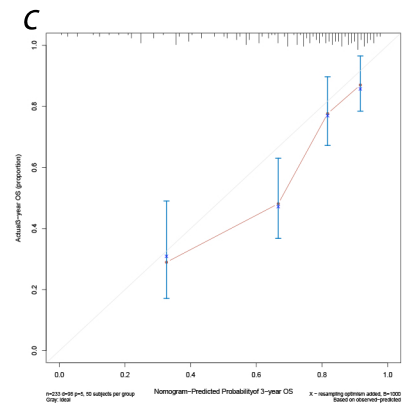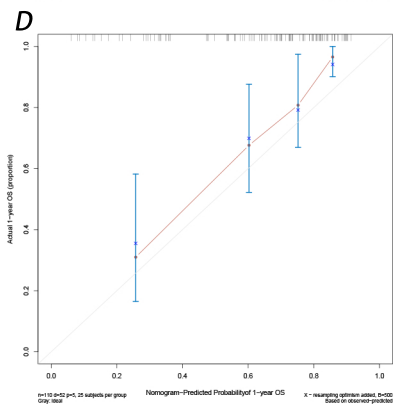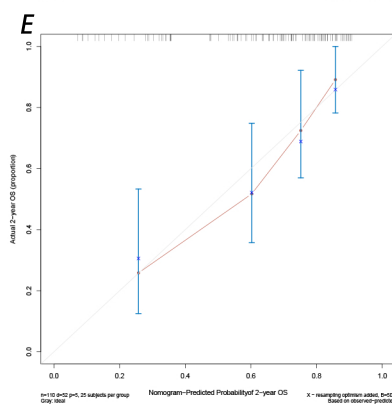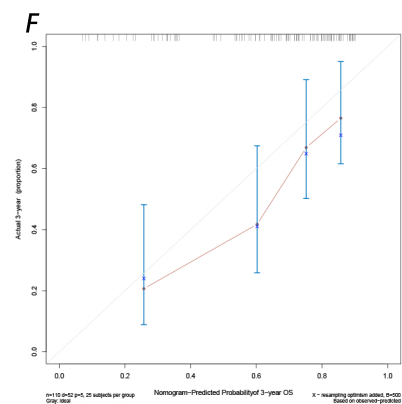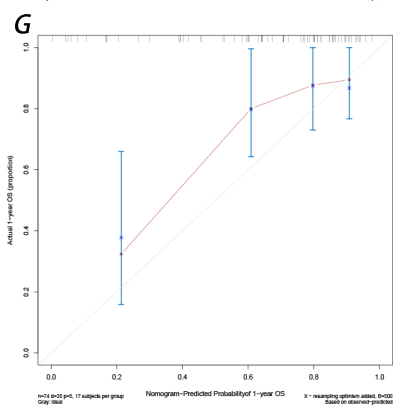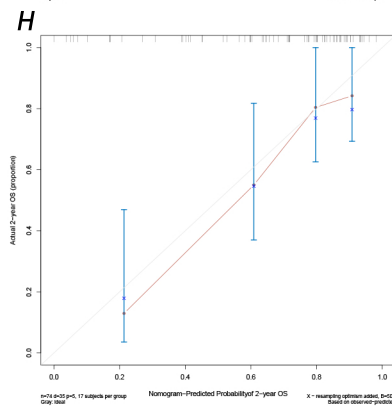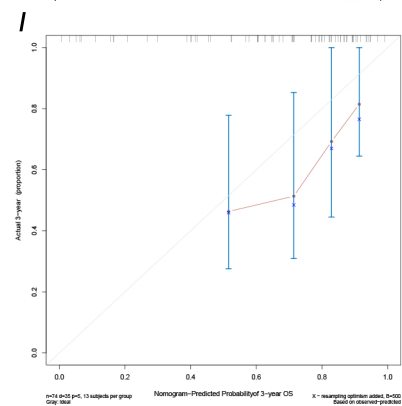

Supplement: Supplementary file 2 — Additional file 2: Supplementary Figure 2. Calibration curves of the nomograms. Calibration curves of 1-, 2- and 3-year overall survival for patients with pancreatic neuroendocrine tumors presenting with liver metastases in the (A–C) training cohort, (D–F) internal validation cohort, and (G–I) external validation cohort. The grey line represents the ideal reference line, where the predicted probability would match the observed survival rate. The blue dots are calculated by bootstrapping (resamples: 1000) and represent the nomogram performance. The closer the solid red line is to the dotted line, the more accurate the model is in predicting overall survival. [file 12885_2023_10893_MOESM2_ESM.pdf]
